# Isolation and Characterization of Cellulose-decomposing Bacteria Inhabiting Sawdust and Coffee Residue Composts

**DOI:** 10.1264/jsme2.ME11299

**Published:** 2012-02-22

**Authors:** Mohamed Fathallh Eida, Toshinori Nagaoka, Jun Wasaki, Kenji Kouno

**Affiliations:** 1 Graduate School of Biosphere Science, Hiroshima University, 4–4 Kagamiyama 1-Chome, Higashi-hiroshima 739–8528, Egypt; 2 Agricultural Microbiology Department, National Research Center, 33 El Behous St., Dokki, 12622 Cairo, Egypt

**Keywords:** AZCL-substrates, bacteria, cellulolytic activity, compost, hemicellulolytic activity

## Abstract

Clarifying the identity and enzymatic activities of microorganisms associated with the decomposition of organic materials is expected to contribute to the evaluation and improvement of composting processes. In this study, we examined the cellulolytic and hemicellulolytic abilities of bacteria isolated from sawdust compost (SDC) and coffee residue compost (CRC). Cellulolytic bacteria were isolated using Dubos mineral salt agar containing azurine cross-linked (AZCL) HE-cellulose. Bacterial identification was performed based on the sequence analysis of 16S rRNA genes, and cellulase, xylanase, β-glucanase, mannanase, and protease activities were characterized using insoluble AZCL-linked substrates. Eleven isolates were obtained from SDC and 10 isolates from CRC. DNA analysis indicated that the isolates from SDC and CRC belonged to the genera *Streptomyces*, *Microbispora*, and *Paenibacillus*, and the genera *Streptomyces*, *Microbispora*, and *Cohnella*, respectively. *Microbispora* was the most dominant genus in both compost types. All isolates, with the exception of two isolates lacking mannanase activity, showed cellulase, xylanase, β-glucanase, and mannanase activities. Based on enzyme activities expressed as the ratio of hydrolysis zone diameter to colony diameter, it was suggested that the species of *Microbispora* (SDCB8, SDCB9) and *Paenibacillus* (SDCB10, SDCB11) in SDC and *Microbispora* (CRCB2, CRCB6) and *Cohnella* (CRCB9, CRCB10) in CRC contribute to efficient cellulolytic and hemicellulolytic processes during composting.

Cellulose, which represents approximately 1.5×10^12^ tons of the annual biomass produced through photosynthesis, is the most abundant organic polymer and is considered to be an almost inexhaustible source of raw materials for various products (44). Furthermore, hemicelluloses, which consist of a heterogeneous group of polysaccharides that includes xylans, β-glucans, and mannans, are also important constituents of plant cell walls (5). The cellulose, hemicellulose, and lignin contents of organic wastes vary depending on the source (19, 35). For example, coffee residue consists of 35% cellulose, 46% hemicellulose, and 19% lignin (40), whereas sawdust typically contains 40%–50% cellulose, 25%–35% hemicellulose, and 20%–30% lignin (43). Due to their abundance, the recycling of lignocellulosic materials is indispensable for the carbon cycle and requires numerous time-consuming processes, which include mechanical, chemical, thermal, and biological treatments (31). Degradation of lignocellulosic materials in their natural environments proceeds exclusively through biological processes (6). Therefore, investigating the microbial communities inhabiting compost and clarifying their role in the biodegradation of organic components is an important step for improving composting processes.

During composting, numerous types of microorganisms influence the biological degradation of wastes. Although fungi are the main cellulase-producing microorganisms, a few species of bacteria and actinomycetes have also been reported to produce cellulase (44, 58) and are involved throughout the degradation process (51). Cellulolytic and hemicellulolytic bacteria have been isolated from a wide diversity of environments, such as soils, composts, decaying plant wastes, and the feces of ruminant animals (30). Bacteria and actinomycetes play specific roles in the biodegradation of organic materials during composting (14, 33).

The biodegradation of waste materials occurs by the concerted action of various microorganisms which produce a series of enzymes that contribute to the bioconversion process (37). These enzymes include cellulases, hemicellulases, and pectinases, which function synergistically to degrade complex cell-wall molecules (9). Most studies of biodegradation processes have emphasized the role of fungi because of their capability of producing and secreting high amounts of enzymes (30). Several methods based on the use of soluble or insoluble substrates have been applied to determine the cellulolytic and hemicellulolytic activities of microorganisms (47). However, most of these methods are not quantitative or sufficiently sensitive due to the poor correlation between enzyme activity and the resulting hydrolysis zone diameter (30). Thus, to more accurately evaluate cellulolytic and hemicellulolytic microorganisms, an efficient plate-screening method is required.

Recently, a few chromogenic substrates have been used for measuring microbial enzymatic activities. In particular, azurine cross-linked (AZCL) substrates are commercially available and have been successfully applied for the evaluation of enzymatic activities (8, 36). During the degradation of AZCL substrates, colored particles are released and form a blue-colored zone around the colony, with the intensity of the colored zone being based on several parameters, including the substrate and enzyme concentrations, and the catalytic properties of the active enzyme. Using AZCL substrates, a positive and quantitative correlation has been demonstrated between enzyme activity and the diameters of the blue zones (8, 36, 48).

In a previous report, we investigated the cellulolytic and hemicellulolytic abilities of fungi isolated from mature sawdust (SDC) and coffee residue composts (CRC). In this study, we evaluated the cellulolytic and hemicellulolytic abilities of bacteria isolated from SDC and CRC in an attempt to clarify the role of these bacteria in the biodegradation of organic wastes during the composting process.

## Materials and Methods

### Isolation of cellulose-decomposing bacteria

SDC produced from the residue of medium used to culture mushrooms was obtained from the Agricultural Cooperative Association of Saitama Prefecture, Japan, and CRC was collected from a composting center located in Hiroshima Prefecture, Japan. The two types of compost were produced by composting heaps of the respective starting materials that were turned periodically during a 2- to 3-month period until reaching maturity. The chemical and biological properties of SDC and CRC are presented in Table 1.

Bacteria were isolated from SDC and CRC using a dilution plate method. Briefly, the primary suspensions were prepared by suspending 10 g of each compost type in 90 mL sterile distilled water, and the resulting suspensions were then shaken at 150 rpm for 30 min at room temperature. Ten-fold serial dilutions were then prepared in sterilized distilled water. One-hundred microliters from the 10^−7^ dilution of each compost type was spread on Dubos mineral salt agar medium (NaNO_3_, 0.5 g L^−1^; K_2_HPO_4_, 1 g L^−1^; MgSO_4_·7H_2_O, 0.5 g L^−1^; FeSO_4_·7H_2_O, 0.02 g L^−1^; KCl, 0.2 g L^−1^; and agar, 20 g L^−1^, pH 7.5) (10), which was supplemented with 0.05% (w/v) AZCL HE-Cellulose (Megazyme, Bray, Ireland) as the sole carbon source. Five plates were prepared for each compost type and were incubated at 35°C for 7 days. Colonies of cellulose-degrading bacteria, which were surrounded by a blue halo zone indicating cellulase activity, were developed on the plates. Each representative plate was selected from SDC and CRC, on which 11 and 10 colonies of cellulose-degrading bacteria appeared, respectively. All colonies of cellulose-degrading bacteria were isolated from each plate and transferred to Trypto-Soya Agar plates (TSA; Nissui Pharmaceutical, Tokyo, Japan). A single colony of each isolate was transferred repeatedly on TSA to obtain a pure culture.

### DNA extraction and PCR amplification

Bacterial cells used for DNA extraction were cultivated on TSA medium for approximately 5 days at 35°C. Genomic DNA was extracted by the boiling method. Briefly, a bacterial colony was picked up from the surface of a TSA plate with a 10-μL micropipette tip and suspended in 25 μL sterile water in a PCR tube. The tubes were heated at 94°C for 5 min using a thermal cycler (GeneAmp PCR System 2700; Applied Biosystems, Foster City, CA, USA), and the extracted DNA solution was used as a template for PCR amplification. PCR was performed with a pair of universal bacterial primers targeting the 16S rRNA gene; 27F (GAGTTTGATCMTG GCTCAG) and 1492R (TACGGYTACCTTGTTACGACTT) as forward and reverse primers, respectively, according to Lane (28). PCR was performed in a 50-μL volume containing 10 ng genomic DNA, 25 μL 2× PCR Buffer for KOD FX Neo, 10 μL dNTP mix (2 mM each), 1 U KOD FX Neo polymerase (Toyobo, Osaka, Japan), and 1.5 μL (10 μM) of each primer (27F and 1492R). After an initial denaturation step at 94°C for 2 min, target DNA was amplified in 30 cycles. Each cycle consisted of denaturation at 98°C for 10 s, annealing at 51°C for 30 s, and extension at 68°C for 90 s. A final extension was performed at 68°C for 5 min. The PCR products were analyzed by agarose gel electrophoresis. After electrophoresis, gels were stained with ethidium bromide and visualized by UV transillumination with the printgraph AE-6932GXCF system (ATTO, Tokyo, Japan). The PCR products were purified using a QIAquick PCR Purification Kit (Qiagen, Hilden, Germany) according to the manufacturer’s instructions.

### Sequencing and data analysis

The 16S rRNA gene sequences of isolated bacteria were determined by direct sequencing of the purified PCR-amplified 16S rDNA fragments. Sequencing was performed using the BigDye Terminator v3.1 Cycle Sequencing Kit (Applied Biosystems) on a 3730xl DNA analyzer (Applied Biosystems). Nearly complete 16S rRNA gene sequences were obtained from each isolate using the following oligonucleotides: 27F, 515F (GTGCCAGCMGCCGCG GTAA) (52), F984 (AACGCGAAGAACCTTAC) (20), and 519R (GWATTACCGCGGCKGCTG) (28).

The obtained 16S rDNA sequences of isolated bacteria were compared with those of other known species deposited in the GenBank database (http://blast.ncbi.nlm.nih.gov/Blast.cgi) using the BLASTN 2.2.24 program (61). A phylogenetic tree based on partial 16S rRNA sequences was constructed using the neighbor-joining method contained within the Clustal X program (50) and MEGA4 software (46).

### Enzyme assays

Dubose mineral agar medium containing 0.05% of an AZCL-substrate (AZCL-HE-cellulose, -arabinoxylan, -barly β-glucan, -galactomannan, or -casein) as the sole carbon source was applied to measure cellulase, xylanase, β-glucanase, mannanase, and protease activities, respectively, of isolated bacteria. To evaluate enzyme activities, bacterial isolates were point-inoculated on AZCL substrate-Dubos agar plate surfaces and were incubated for 7 days at 35°C. Enzymatic activity was expressed as the Substrate Hydrolysis Index, which was calculated as the ratio between the average diameter (d) of the hydrolysis zone of substrate (the blue zone around the bacterial colony) and the average diameter of the bacterial colony (D) in millimeters (8).

### Nucleotide sequence accession numbers

The nucleotide sequences determined in this study have been deposited in GenBank under nucleotide accession numbers JN617210–JN617230.

## Results

### Isolation and phylogenetic analysis of bacterial isolates

The two compost types, SDC and CRC, were subjected to the isolation of cellulolytic bacteria, whose cellulolytic and hemicellulolytic activities were subsequently characterized. All colonies of cellulose-degrading bacteria surrounded by a blue halo zone were isolated from each plate of SDC and CRC, selected from 5 replicate plates. The 16S rRNA gene sequence analyses of the 21 bacterial isolates revealed that they were closely related to four genera: *Streptomyces*, *Microbispora*, *Paenibacillus*, and *Cohnella*. The genera *Streptomyces* and *Microbispora* were dominant in both compost types, whereas *Paenibacillus* was only obtained from SDC and *Cohnella* was only isolated from CRC (Table 2).

**Fig. 1:**
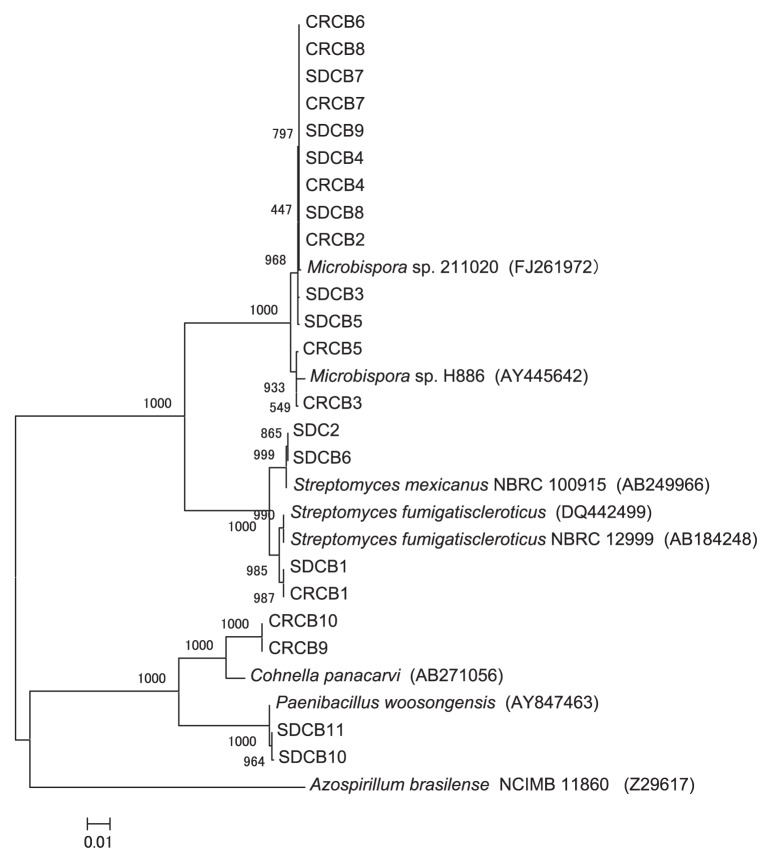
Neighbor-joining tree showing the relationship between the 16S rRNA gene sequences from bacteria isolated from SDC and CRC. Bootstrap values of neighbor-joining analysis from 1,000 replications are shown on the branches. Scale bar represents the number of changes per nucleotide position (substitution/site).

With regard to the SDC bacterial isolates, BLAST search results and the constructed phylogenetic tree based on the 16S rRNA gene sequence data (Fig. 1) showed that three isolates (SDCB1, SDCB2, and SDCB6) were closely related (>99% similarity) to two different species of the genus *Streptomyces*. Six of the 11 isolates (54.5%) displayed >99% similarity to members of the genus *Microbispora*, while the remaining two isolates (SDCB10 and SDCB11) matched the genus *Paenibacillus*, with 99.7% maximum similarity to *Paenibacillus woosongensis* (Table 2).

Concerning the CRC bacterial isolates, BLAST search results and the constructed 16S rRNA phylogenetic tree revealed that one isolate (CRCB1) showed 99.4% similarity to *Streptomyces fumigatiscleroticus*, while seven isolates displayed ≥99% similarity to the genus *Microbispora*. Furthermore, two isolates (CRCB9 and CRCB10) exhibited relatively low similarity (96.1% and 96.2%, respectively) to *Cohnella panacarvi* (Table 2 and Fig. 1).

**Fig. 2:**
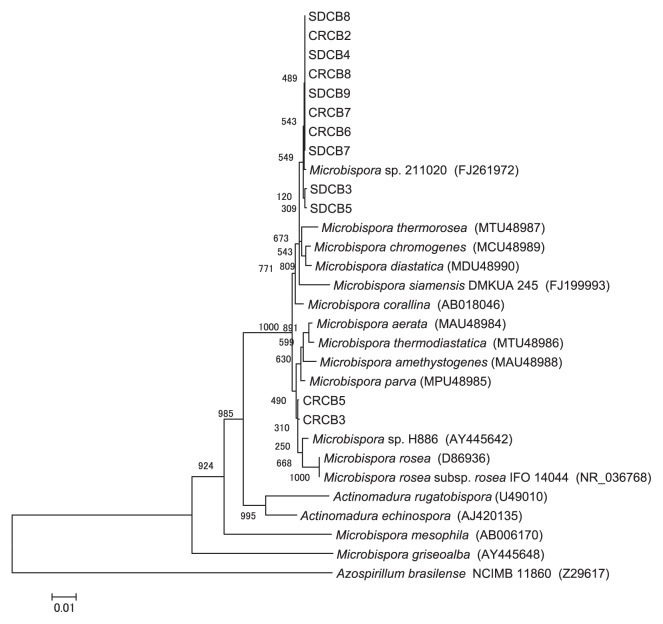
Neighbor-joining tree showing the relationship between the 16S rRNA gene sequences from *Microbispora* sp. isolates and the closest genera and species deposited in the GenBank database. Bootstrap values of neighbor-joining analysis from 1,000 replications are shown on the branches. Scale bar represents the number of changes per nucleotide position (substitution/site).

Based on the BLAST results, none of the *Microbispora* isolates could be identified to the species level due to a lack of high similarity to species within the genus. Thus, the phylogenetic relationship between the 16S rRNA sequences of the *Microbispora* isolates and those of *Microbispora* species deposited in the GenBank database were further examined. The constructed phylogenetic tree showed that two isolates (CRCB3 and CRCB5) were closely related to *Microbispora rosea* subsp. *rosea* (AY445647), whereas the other *Microbispora* isolates were not clearly matched to any known species of *Microbispora* (Fig. 2); however, these *Microbispora* isolates were contiguous with *Microbispora thermorosea* (MTU48987), *M. chromogenes* (MCU48989), and *M. diastatica* (MDU48990), as illustrated in Fig. 2.

**Fig. 3:**
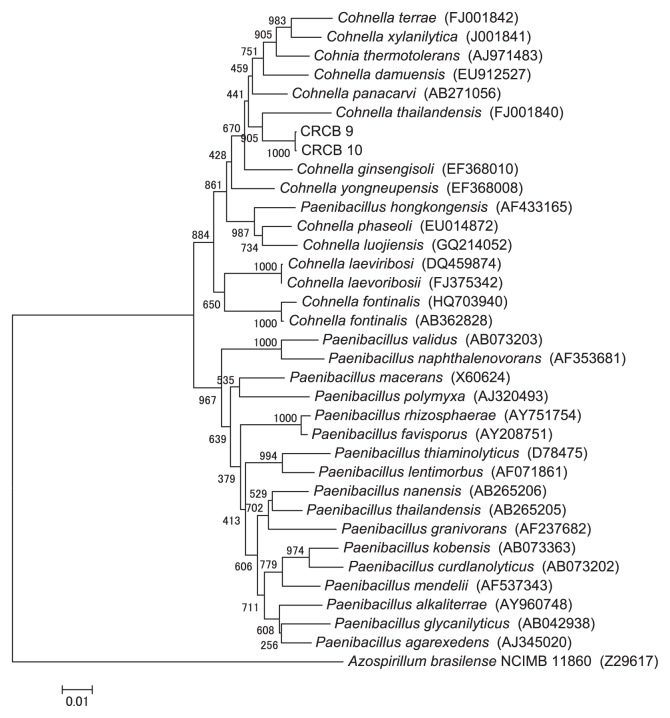
Neighbor-joining tree showing the relationship between the 16S rRNA gene sequences from *Cohnella* sp. and the species of the genera *Cohnella* and *Paenibacillus*. Bootstrap values of neighbor-joining analysis from 1,000 replications are shown on the branches. Scale bar represents the number of changes per nucleotide position (substitution/site).

The CRC isolates CRCB9 and CRCB10 displayed comparatively low similarity (96%) to genus *Cohnella*, although the BLAST search indicated that these isolates also had similarity to *Paenibacillus* spp. More detailed phylogenetic analysis of the CRCB9 and CRCB10 16S rRNA gene sequences and of species of the genera *Cohnella* and *Paenibacillus* showed that both CRCB9 and CRCB10 were closer to the genus *Cohnella* than the genus *Paenibacillus* (Fig. 3). The top BLAST result showed that the closest species to CRCB9 and CRCB10 was *Cohnella panacarvi* (AB271056), while the constructed tree revealed that both isolates were closest to *Cohnella thailandensis* (FU001840).

### Characterization of SDC isolates

The three *Streptomyces* isolates showed cellulase, xylanase, β-glucanase, mannanase, and protease activities (Table 3). The isolate closest to *S. fumigatiscleroticus* (SDCB1) showed higher cellulase, β-glucanase, and mannanase activities than *Streptomyces mexicanus* (SDCB2 and SDCB6); however, the SDCB2 and SDCB6 isolates displayed higher xylanase and protease activities than the SDCB1 isolate.

All six *Microbispora* isolates displayed activities in the five enzyme assays; however, the ability of the *Microbispora* isolates to degrade the various substrates varied among the different isolates. Notably, the *Microbispora* isolates showed high cellulase, xylanase, mannanase, and protease activities; isolate SDCB9 showed the highest cellulase activity, SDCB8 had the highest xylanase and mannanase activities, and SDCB3 exhibited the highest protease activity among the SDC isolates.

The two *Paenibacillus* isolates exhibited cellulase, xylanase, β-glucanase, and mannanase activities, but protease activity was not detected. The highest β-glucanase activity among the SDC isolates was displayed by the *Paenibacillus* isolates, particularly SDCB10. In addition, the *Paenibacillus* isolates exhibited relatively high cellulase and mannanase activities.

### Characterization of CRC isolates

*Streptomyces* isolate CRCB1, which was phylogenetically close to *S. fumigatiscleroticus*, showed relatively low cellulase activity and the lowest xylanase, β-glucanase, and protease activities among the CRC isolates.

The *Microbispora* isolates, with the exception of CRCB2, showed degradative activity towards all of the AZCL substrates used for the enzyme assays. Although the CRCB2 isolate did not have the ability to degrade AZCL-galactomannan, it exhibited the highest cellulase activity. The CRCB6 isolate showed the highest xylanase and β-glucanase activities, while CRCB5 and CRCB2 exhibited the highest mannanase and protease activities, respectively, among the *Microbispora* isolates from CRC.

The two isolates closest to *Cohnella* sp. (CRCB9 and CRCB10) displayed the highest mannanase and xylanase activities among the CRC isolates, and relatively high cellulase activity among all compost isolates, although protease activity was not detected.

## Discussion

In the present study, we investigated the cellulolytic and hemicellulolytic activities of bacteria isolated from SDC and CRC in an effort to elucidate their microbial capability to degrade lignocellulosic materials. In both SDC and CRC, *Streptomyces* spp. and *Microbispora* spp. were the dominant isolates. Our enzymatic analysis revealed that *Microbispora* sp. isolate SDCB8 displayed high xylanase and mannanase activities, and SDCB9 and SDC11 indicated the highest cellulase and β-glucanase activities, respectively, in SDC, while CRCB2, CRCB6, CRCB9, and CRCB10 showed the highest cellulase, β-glucanase, mannanase, and xylanase activities, respectively, in CRC.

The isolated genera from SDC consisted of *Streptomyces* (3 isolates), *Microbispora* (6 isolates), and *Paenibacillus* (2 isolates), while *Streptomyces* (1 isolate), *Microbispora* (7 isolates), and *Cohnella* (2 isolates) were isolated from CRC. The 16S rRNA phylogenetic analysis revealed that of the four *Streptomyces* isolates, one was a close relative of *S. fumigatiscleroticus*, which was present in both compost types, while a second SDC isolate was a close relative of *S. mexicanus* (Table 1 and Fig. 1). Our findings are consistent with those of Kleyn and Wetzler (27), who isolated several species of *Streptomyces* from spent mushroom compost. Adhikary *et al.*(1) also found that *Streptomyces* spp. were predominant at all stages of the composting of mushroom components, paddy straw, sugar cane bagasse, and water-hyacinth.

Although our analysis showed that many isolates from both compost types belonged to the genus *Microbispora* (54.6% and 70.0% of SDC and CRC isolates, respectively), El-Tarabily and Sivasithamparam (13) reported that the genus *Microbispora* represented only 0.18% of total actinomycetes isolated from soil. Although Waldron *et al.*(56) isolated *Microbispora* from several soil samples, streams, pools, and compost samples, there are no other reports of *Microbispora* inhabiting compost. The high population of *Microbispora*-related isolates identified in both compost types examined here suggests that these strains are well adapted to composting conditions.

Two SDC isolates were identified as members of the genus *Paenibacillus* and displayed 99.7% 16S rRNA sequence similarity to *P. woosongensis. Paenibacillus* spp. have also been isolated from municipal urban waste compost (54), poultry litter compost (53), cow feces (55), and spent mushroom compost (57). We also found that two CRC isolates showed similarity (approx. 96%) to *Cohnella* sp. The low similarity of these isolates to known species of the genus *Cohnella* suggests that they may represent new species. The classification of these isolates by other taxonomic methods, such as biochemical analysis, fatty acids analysis, DNA base composition, and DNA-DNA hybridization, is required to confirm this speculation; however, our present finding are supported by Rastogi *et al.*(38), who reported that *Cohnella* is one of the dominant cellulolytic bacterial genera isolated from compost samples.

The microbial enzymatic activities clearly varied among both the isolated genera and between related isolates. El-Refai *et al.*(12) also observed variations in bacterial and fungal enzyme activities among species of the same genus and even between different strains of the same species. Béra-Maillet *et al.*(3) revealed that cellulase and hemicellulase activities differed among strains of the genus *Fibrobacter*.

Among SDC bacteria isolates, the three *Streptomyces* isolates (SDCB1, SDCB3, and SDCB6) hydrolyzed AZCL-cellulose, -arabinoxylan, -β-glucan, -galactomannan, and -casein. Several species of *Streptomyces* are known to produce cellulase enzymes (9), and a number of *Streptomyces* strains have been reported to produce cellulase and xylanase (8). Grabski and Jeffries (17) demonstrated that *Streptomyces* sp. possess xylanase, and Théberge *et al.*(49) isolated and characterized endoglucanase from the culture filtrate of *Streptomyces* sp. Takahashi *et al.*(45) found that galactomannan was hydrolyzed by a purified extracellular mannanase from *Streptomyces* sp. Kansoh and Nagieb (23) reported that xylanase and mannanase were produced by *Streptomyces* sp. cultivated in a medium containing xylan from either sugar cane bagasse or galactomannan. Additionally, a strain of *Streptomyces* was shown to overproduce the extracellular protease against the substrate azocasein (16). Based on our present findings and those of previous studies, the isolates closest to *Streptomyces* may be important contributors to the degradation of the different components of organic materials during the composting process.

*Microbispora* isolates showed the highest cellulase activity (SDCB9), xylanase and mannanase activities (SDCB8), and protease activity (SDCB3) in SDC. Waldron *et al.*(56) demonstrated that *Microbispora* produces thermally stable extracellular cellulase in good yield, while the poor xylanase activity of *Microbispora* sp. isolated from cattle manure was reported by Holtz *et al.*(21). Here, all *Microbispora* isolates were able to degrade AZCL-β-glucan as a substrate for β-glucanase activity. In accordance with our results, Bartley *et al.*(2) detected and purified β-glucanase produced by *Microbispora* sp. Although our *Microbispora* sp. isolates showed high mannanase activity, to our knowledge, no previous reports have identified mannanase production by *Microbispora* sp. Furthermore, the *Microbispora* isolates showed higher protease activity than the other genera isolated from SDC, a finding that contrasts with that of Nawani *et al.*(34), who found that *Microbispora* sp. displayed very low protease activity. This discrepancy may be due to variations in the enzyme profiles of microbial isolates among different species of the same genus, as well as among different isolates of the same species.

Isolates SDCB10 and SDCB11 were closely related to *P. woosongensis* and showed cellulase, xylanase, mannanase, and the highest β-glucanase activities, although protease activity was not detected. Rivas *et al.*(39) also found that cellulases, xylanases, and β-galactosidase were actively produced by a *Paenibacillus* sp. isolated from the phyllosphere of *Phoenix dactylifera*, but caseinase was not produced. In addition, Shi *et al.*(41) reported the production of a novel bifunctional xylanase by *Paenibacillus* sp. with xylanase and glucanase activities, and Fu *et al.*(15) demonstrated the mannanase activity of *Paenibacillus* sp. Thus, isolates closest to *Paenibacillus* sp. may play a significant role in the degradation of cellulose, xylans, and mannans of SDC during the composting process.

With regard to the bacteria isolated from CRC, one isolate (CRCB1) showed 99.4% similarity to *S. fumigatiscleroticus* and possessed cellulase, xylanase, β-glucanase, and protease activities, but no detectable mannanase activity. Grabski and Jeffries (17) also demonstrated the production of cellulase and xylanase by *S. fumigatiscleroticus*. Malviya *et al.*(32) reported that a number of *Streptomyces* isolates produce cellulases and proteases, while Hong *et al.*(22) revealed that β-glucanase is produced by *Streptomyces* sp. Although *Streptomyces* isolates closely related to *S. fumigatiscleroticus* (SDCB1) from SDC revealed mannanase activity, CRCB1 (*S. fumigatiscleroticus*) could not degrade galactomannan. This finding may indicate that the enzyme profile of microorganisms is not only genus and species-specific, but also isolate-specific. This speculation is supported by the result of our previous study, in which we also found that the enzyme profiles of cellulolytic and hemicellulolytic fungi were isolate-specific (11).

As found in SDC, *Microbispora* sp. was the predominant genus detected in CRC. All *Microbispora* isolates displayed activities in all five of the enzyme assays with the exception of CRCB2, which showed the highest cellulase activity, but did not exhibit mannanase activity. Although cellulase (56), xylanase (21), β-glucanase (2), and protease activities (34) have been identified among *Microbispora* sp., there are no reports of mannanase activity by members of this genus, as described above. Although no previous studies concerning the role of *Microbispora* sp. in composting processes were found, the results of the present study suggest that this genus may play an important role in the biodegradation of organic material.

The isolates closely related to *C. panacarvi* (CRCB9 and CRCB10) displayed cellulase, xylanase, β-glucanase, and mannanase activities. Notably, CRCB9 and CRCB10 exhibited the highest mannanase and xylanase activities, respectively among the ten CRC isolates. Rastogi *et al.*(38) found that a *Cohnella* sp. was able to degrade cellulose, carboxymethyl cellulose (CMC), and sawdust. Xylanolytic activity of the genus *Cohnella* was also reported for several species, including *C. panacarvi* sp. (60), *C. damensis* sp. (59), and *C. thailandensis* sp. (26). In agreement with our results, Cho *et al.*(7) demonstrated that *Cohnella* sp. isolates were negative for protease activity; however, no previous reports concerning the β-glucanase and mannanase activities of any members of the genus *Cohnella* were found. CRCB9 and CRCB10 isolates may contribute to the effective degradation of xylans and mannans, and may also be important for the degradation of cellulose during the composting of CRC.

In this study, aerobic cellulolytic bacteria were isolated and their enzyme activities evaluated. Some anaerobic bacteria, such as *Clostridium* spp. are known to degrade cellulose and hemicellulose. Sizova *et al.*(42) isolated cellulose- and xylan-degrading anaerobic bacteria, *Clostridium* spp., from biocompost made from wood chips, horse manure, and food waste. Haruta *et al.*(18) constructed a microbial community with high cellulolytic ability by composting materials consisting of rice straw, sugar cane dregs, chicken and cattle feces, while Kato *et al.*(24) isolated a novel anaerobic bacterium, *Clostridium straminisolvens*, as a cellulolytic bacterium from their microbial community. Their study under aerobic conditions using a mixed culture composed of five bacteria strains, *C. straminisolvens*, *Clostridium* sp., *Pseudoxanthomonas* sp., *Brevibacillus* sp., *Bordetella* sp., which were isolated from their microbial community, demonstrated synergistic relationships between an anaerobic cellulolytic bacterium (*C. straminisolvens*) and aerobic bacteria such as *Pseudoxanthomonas* sp. and *Brevibacillus* sp. It was suggested that aerobes serve to create anaerobic conditions through their respiration activities, while anaerobic cellulolytic bacteria supply substrates such as acetate and glucose for aerobes (25). Anaerobic Clostridia bacteria were also detected in rice straw-composting materials and in aerated cattle manure composting piles by PCR-DGGE (4, 29). The coexistence of various types of bacteria including cellulolytic and hemicellulolytic anaerobes could be associated with composting processes. Isolation of both cellulose-degrading aerobic and anaerobic bacteria from composts and evaluation of their cellulolytic and hemicellulolytic enzyme activities, however, have not been reported.

In conclusion, all SDC and CRC isolates among the four identified genera likely cooperated in the degradation of celluloses, hemicelluloses, and proteins based on the observed enzyme profiles. Although no previous investigations have reported the role of *Microbispora* in the degradation of organic materials during composting, the present study suggests the importance of this genus in the composting process. The *Microbispora* isolates identified here may play a major role in the degradation process in both examined compost types. Our findings indicate that *Microbispora* (SDCB8 and SDCB9) and *Paenibacillus* isolates (SDCB10 and SDCB11) may contribute to effective biodegradation in SDC, whereas *Microbispora* (CRCB2 and CRCB6) and *Cohnella* isolates (CRCB9 and CRCB10) could be important for the enhancement of cellulose and hemicellulose bioconversion during the composting of CRC. To our knowledge, the present study is the first report to describe the production of mannanase enzymes by *Microbispora* sp. and to identify β-glucanase and mannanase activities within the genus *Cohnella*. This study examined only aerobic cellulose-degrading bacteria and their enzyme activities. Further study should be conducted to evaluate the contribution of anaerobic cellulose-degrading bacteria during composting.

## Figures and Tables

**Table 1:** Chemical and biological properties of SDC and CRC

Compost type	Dry matter (g kg^−1^)	Total (g kg^−1^)‡			C/N ratio	Cellulose degrading bacteria (×10^8^ cfu g^−1^)‡

		C	N	P		
SDC	538	456	15	12	30.0	11.7
CRC	390	448	21	0.7	20.9	9.9

‡ Values expressed on an oven-dry basis.

SDC: sawdust compost.

CRC: coffee residue compost.

**Table 2:** Top BLAST results for bacterial 16S rRNA sequences showing GenBank accession numbers of bacteria isolated from SDC and CRC

Isolate No.	GenBank accession No.	Top BLAST hit GenBank	Similarity (%)
SDCB1	JN617210	*Streptomyces fumigatiscleroticus* NRRL B-3856T (DQ442499)	99.2
SDCB2	JN617211	*Streptomyces mexicanus* NBRC 100915 (AB249966)	99.9
SDCB3	JN617212	*Microbispora* sp. 211020 (FJ261972)	99.7
SDCB4	JN617213	*Microbispora* sp. 211020 (FJ261972)	99.8
SDCB5	JN617214	*Microbispora* sp. 211020 (FJ261972)	99.7
SDCB6	JN617215	*Streptomyces mexicanus* NBRC 100915 (AB249966)	99.9
SDCB7	JN617216	*Microbispora* sp. 211020 (FJ261972)	99.8
SDCB8	JN617217	*Microbispora* sp. 211020 (FJ261972)	99.8
SDCB9	JN617218	*Microbispora* sp. 211020 (FJ261972)	99.8
SDCB10	JN617219	*Paenibacillus woosongensis* YB-45 (AY847463)	99.7
SDCB11	JN617220	*Paenibacillus woosongensis* YB-45 (AY847463)	99.7

CRCB1	JN617221	*Streptomyces fumigatiscleroticus* NBRC 12999 (AB184248)	99.4
CRCB2	JN617222	*Microbispora* sp. 211020 (FJ261972)	99.8
CRCB3	JN617223	*Microbispora* sp. H886 (AY445642)	99.6
CRCB4	JN617224	*Microbispora* sp. 211020 (FJ261972)	99.8
CRCB5	JN617225	*Microbispora* sp. H886 (AY445642)	99.6
CRCB6	JN617226	*Microbispora* sp. 211020 (FJ261972)	99.7
CRCB7	JN617227	*Microbispora* sp. 211020 (FJ261972)	99.8
CRCB8	JN617228	*Microbispora* sp. 211020 (FJ261972)	99.8
CRCB9	JN617229	*Cohnella panacarvi* (AB271056)	96.1
CRCB10	JN617230	*Cohnella panacarvi* (AB271056)	96.2

SDCB: bacteria isolated from saw dust compost; CRCB: bacteria isolated from coffee residue compost. Numbers showed the isolate number.

**Table 3:** Enzyme activities of cellulolytic bacteria isolated from SDC and CRC

Genus	Isolate No.	Hydrolysis index				

		cellulase	xylanase	β-glucanase	mannanase	protease
*Microbispora*	SDCB3	4.2 ± 0.7	15.0 ± 4.0	12.9 ± 5.4	9.3 ± 1.3	**11.8** ± 2.5
	SDCB4	4.9 ± 0.5	12.1 ± 4.0	8.1 ± 0.7	8.2 ± 1.3	9.9 ± 1.8
	SDCB5	5.2 ± 1.1	17.2 ± 7.1	11.6 ± 2.7	9.8 ± 2.2	7.0 ± 2.0
	SDCB7	5.1 ± 0.9	14.9 ± 4.5	11.6 ± 5.0	7.5 ± 1.2	5.9 ± 0.7
	SDCB8	4.0 ± 1.1	**28.4** ± 5.9	14.8 ± 1.9	**13.4** ± 2.7	11.3 ± 1.7
	SDCB9	**7.7** ± 2.0	9.4 ± 0.9	10.0 ± 2.6	10.5 ± 1.2	4.9 ± 2.3

*Streptomyces*	SDCB1	3.9 ± 0.4	9.0 ± 0.6	9.4 ± 3.4	10.6 ± 2.3	3.5 ± 0.2
	SDCB2	2.7 ± 0.3	9.8 ± 1.4	7.0 ± 0.8	7.2 ± 0.6	4.4 ± 0.7
	SDCB6	3.5 ± 0.7	9.7 ± 1.9	8.1 ± 2.3	7.0 ± 1.5	4.4 ± 0.3

*Paenibacillus*	SDCB10	4.1 ± 1.5	5.6 ± 1.6	**23.7** ± 4.3	6.8 ± 0.9	N.D.
	SDCB11	5.0 ± 1.1	7.8 ± 1.4	16.3 ± 1.4	7.3 ± 1.2	N.D.

*Microbispora*	CRCB2	**9.1** ± 2.9	9.8 ± 2.9	11.1 ± 1.6	N.D.	**9.3** ± 1.4
	CRCB3	3.3 ± 0.5	11.3 ± 1.7	14.0 ± 3.7	9.1 ± 2.2	5.9 ± 1.2
	CRCB4	4.4 ± 0.4	8.4 ± 1.0	8.3 ± 4.3	4.6 ± 0.5	6.1 ± 0.7
	CRCB5	2.7 ± 0.4	7.7 ± 0.6	10.3 ± 2.5	11.5 ± 4.0	4.9 ± 1.0
	CRCB6	2.5 ± 0.5	15.9 ± 2.2	**14.4** ± 3.4	8.1 ± 2.0	5.3 ± 1.0
	CRCB7	2.3 ± 0.1	9.9 ± 0.6	10.1 ± 1.5	11.2 ± 4.0	9.0 ± 2.2
	CRCB8	3.9 ± 0.8	14.7 ± 0.8	10.8 ± 2.6	8.3 ± 1.5	7.3 ± 0.9

*Streptomyces*	CRCB1	3.1 ± 0.6	5.8 ± 1.1	4.7 ± 0.3	N.D.	2.7 ± 0.1

*Cohnella*	CRCB9	6.1 ± 0.9	14.4 ± 0.6	9.8 ± 0.9	**14.2** ± 2.8	N.D.
	CRCB10	6.1 ± 1.2	**17.3** ± 3.3	6.5 ± 0.5	9.2 ± 0.3	N.D.

Enzyme activities expressed as the Substrate Hydrolysis Index, which was calculated as the ratio of the average diameter (d) of the blue zone around the bacterial colony to the average diameter of the bacterial colony (D), expressed in millimeters with standard deviation in five enzyme assays using AZCL-HE-cellulose (cellulase), AZCL-arabinoxylan (xylanase), AZCL-barley β-glucan (β-glucanase), AZCL-galactomannan (mannanase) and AZCL-casein (protease). N.D.: not detected.
